# Single-molecule FRET of protein structure and dynamics - a primer

**DOI:** 10.1186/1477-3155-11-S1-S2

**Published:** 2013-12-10

**Authors:** Benjamin Schuler

**Affiliations:** 1 Department of Biochemistry, University of Zurich, Winterthurerstrasse 190, 8057 Zurich, Switzerland

**Keywords:** Single-molecule spectroscopy, fluorescence, Förster resonance energy transfer, correlation functions, fluorescence correlation spectroscopy, confocal detection, protein dynamics, protein folding

## Abstract

Single-molecule spectroscopy has developed into a widely used method for probing the structure, dynamics, and mechanisms of biomolecular systems, especially in combination with Förster resonance energy transfer (FRET). In this introductory tutorial, essential concepts and methods will be outlined, from the FRET process and the basic considerations for sample preparation and instrumentation to some key elements of data analysis and photon statistics. Different approaches for obtaining dynamic information over a wide range of timescales will be explained and illustrated with examples, including the quantitative analysis of FRET efficiency histograms, correlation spectroscopy, fluorescence trajectories, and microfluidic mixing.

## Introduction

Single-molecule spectroscopy has become an integral part of biophysical research and nanobiotechnology, and a wide range of biological questions are now addressed with these methods [1], including the mechanisms of molecular machines [2-4], protein-nucleic acid interactions [5,6], enzymatic reactions [7-9], and protein or RNA folding [10,11], to name but a few. A key advantage of single-molecule experiments is the possibility to avoid ensemble averaging. Instead of obtaining observables that constitute an average over a large number of molecules in the sample, as it is the case with most conventional methods, information is extracted from the molecules one by one. This often allows direct access to the distributions of the underlying molecular properties, e.g., the number of thermodynamic states populated and the distributions of intramolecular distances or stoichiometries. Another advantage of single-molecule methods is that dynamic information can often be obtained from equilibrium measurements, e.g. via correlation functions [12-16]; the analysis of broadening and exchange between subpopulations in Förster resonance energy transfer (FRET) efficiency histograms [17-20]; or directly from fluorescence trajectories of immobilized molecules [16,19,21-26].

Since the use of these methods has been spreading rapidly, a wide range of literature has become available on the topic, including several textbooks that describe the technical and conceptual details of single-molecule spectroscopy [1,27,28]. Here, I will briefly summarize the key aspects of single-molecule fluorescence spectroscopy on an introductory level, with a focus on the investigation of protein structure and dynamics with single-molecule FRET. I will assume basic knowledge of fluorescence spectroscopy [29] and biomolecular structure and function; beyond that, the principles will be covered step by step, with the goal of familiarising the reader with the type of information available from single-molecule experiments, including potential pitfalls and limitations, and illustrating the capabilities of the method.

This article is based on a tutorial held by the author at the CNRS School "Nanophysics for Health" in November 2012. The examples used to illustrate the concepts are thus primarily taken from the research of the author. For more representative overviews of the wide range of current applications and recent advances in single-molecule spectroscopy, I refer the reader to more comprehensive recent reviews [6,11,30-32].

## From ensembles to single molecules

### Förster resonance energy transfer (FRET)

**Figure 1:**
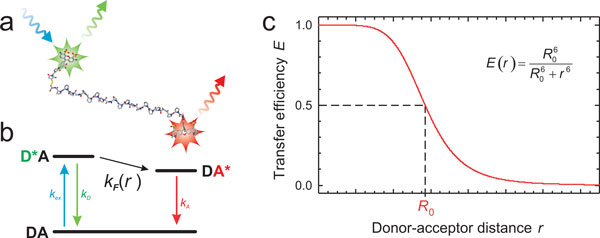
**Förster resonance energy transfer (FRET)**. (a) A molecule labelled with donor and acceptor chromophores (in this case a stiff polyproline peptide with Alexa Fluors 488 and 594 [86]). The donor fluorophore (green) can be excited specifically (blue). It can then either emit a fluorescence photon itself or transfer its excitation energy to the acceptor (red). (b) The process can be depicted in terms of a Jablonsky diagram illustrating the transitions between ground and excited states of donor (D) and acceptor (A). The rate of energy transfer, *k*_F_, depends on the distance, *r*, between donor and acceptor, resulting in the characteristic distance dependence of the transfer efficiency *E *(c). At the Förster distance, *R*_0_, *E *= 0.5. For currently available FRET pairs suitable for single molecule spectroscopy, *R*_0 _is in the range of 4 to 8 nm.

FRET is an attractive method for probing distances and distance dynamics in and between biological macromolecules (such as proteins and nucleic acids) because it is most sensitive in the range of a few nanometres, the typical size of these cellular machines. The theoretical basis of this process was developed already in the 1940s. Theodor Förster [33,34] showed that the rate of excitation energy transfer, *k*_F_, between a suitable donor and acceptor chromophore is proportional to the inverse 6^th ^power of the distance separating them,

(1) $$k_{F}=k_{D}{\left(\frac{R_{0}}{r}\right)}^{6},$$

where *k*_D_^-1 ^is the excited state lifetime of the donor in the absence of the acceptor (Figure 1); *r *is the distance between donor and acceptor; and *R*_0_, the Förster radius, is a proportionality constant that depends on the interaction between the transition dipoles of donor and acceptor. *R*_0 _is calculated as

(2) $$R_{0}^{6}=\frac{9000\;\left(\ln10\right)\;\kappa^{2}Q_{D}J}{128\;\pi^{5}n^{4}N_{A}},$$

where *J *is the overlap integral between the donor emission and the acceptor absorption spectra; *Q*_D _is the donor fluorescence quantum yield; *n *is the refractive index of the medium between the dyes; and *N*_A _is Avogadro's constant. *κ*^2 ^depends on the relative orientation of the chromophores, with

(3) $$\kappa^{2}\;\;\;=\;\;\;{\left(\cos\theta_{T\;\;}\;-\;\;3\;\cos\theta_{D\;}\;\cos\theta_{A\;}\right)}^{2},$$

where *θ*_*T *_is the angle between the donor and acceptor transition dipoles, and *θ*_*D *_and *θ*_*A *_are the angles between the transition moments and the line connecting the centres of donor and acceptor, respectively. In cases where the rotational reorientation of the chromophores is fast compared to the donor excited state lifetime, *κ*^2 ^averages to a value of 2/3, which simplifies the application of FRET significantly. The probability that a photon absorbed by the donor will lead to energy transfer to the acceptor, called the FRET efficiency, *E*, is given by *k*_F_/(*k*_F _+ *k_D_*), which is determined experimentally either by counting the number of emitted donor and acceptor photons or by measuring the donor lifetime in the presence and absence of acceptor (see below). The beauty of Förster theory is that *R*_0 _can be directly obtained, without any theoretical calculations, from readily measurable spectroscopic quantities.

One of the first quantitative tests of Förster's theory and the key experiment that established FRET as a method for the investigation of biomolecules was published by Stryer and Haugland in 1967 [35]. They found the dependence of the transfer efficiency on the inter-chromophore distance to be in agreement with Förster's famous result [33], according to which (Figure 1)

(4) $$E\left(r\right)=\frac{R_{0}^{6}}{R_{0}^{6}+r^{6}}.$$

Consequently, the Förster radius is the characteristic distance that results in a transfer efficiency of 1/2. The idea of this "spectroscopic ruler" [35] has had a large impact on the investigation of biomolecular structure and dynamics on distances in the range of about 1 to 10 nm [29,36-38]. Initially, all of these measurements were done in "ensemble experiments", i.e. by interrogating samples with large numbers of molecules in steady-state or time-resolved fluorometers. About 30 years after the work of Stryer and Haugland, the first single-molecule FRET measurements were reported by Ha *et al*. [39]. But what does it take to do single-molecule fluorescence and FRET experiments?

### Basic considerations

Already in the 1970s, it became possible to measure fluorescence from single atoms in dilute atomic beams [40], i.e. in the gas phase, where the background problem is minimal. Observing single molecules in the condensed phase is much more challenging because Rayleigh and Raman scattering result in a large background signal. Additionally, the ubiquity of contaminants makes great demands on the purity of the matrix. Finally, an atom in vacuum is a chemically very stable system, even in its excited state, whereas fluorescent molecules in the condensed phase survive only a limited number of excitation-emission cycles before they are irreversibly destroyed, typically by a chemical reaction with other molecules, in a process termed photobleaching. Single molecule detection in a solid or liquid matrix (an aqueous solution in the case of most biomolecular experiments) therefore requires additional measures. First of all, the method must provide a signal from an individual molecule that exceeds the background caused by the large number of solvent molecules.

**Figure 2:**
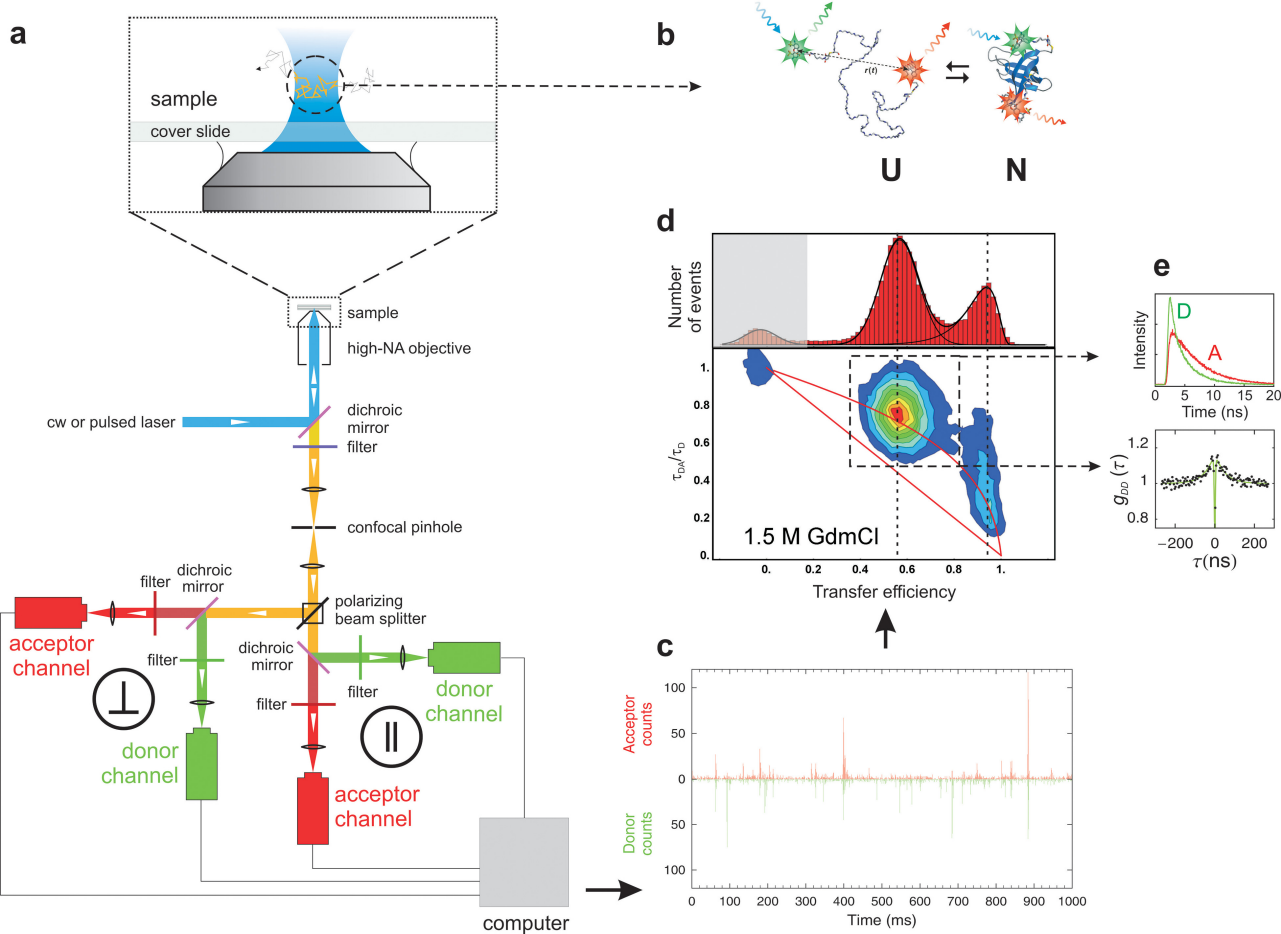
**Overview of instrumentation and data reduction in confocal single-molecule spectroscopy**. (a) The scheme illustrates the main components of a 4-channel confocal single molecule instrument that collects fluorescence photons separated by polarization and wavelength and records their individual arrival times. (b) Illustration of a FRET-labelled protein present in solution in an equilibrium between folded and unfolded states. (c) Small part of a binned trajectory of detected photons recorded from molecules freely diffusing in solution (in this example Csp*Tm *in 1.5 M GdmCl [13]), where each fluorescence burst corresponds to an individual molecule traversing the diffraction-limited confocal volume. (d) Transfer efficiency histogram and 2-dimensional histogram of the relative donor fluorescence lifetime, *τ*_*DA*_/*τ*_D_, versus transfer efficiency, *E*, calculated from individual bursts, resulting in subpopulations that can be assigned to the folded and unfolded protein, and molecules without active acceptor at *E *≈ 0 (shaded in grey). The straight and curved red lines correspond to the relation between *τ*_DA_/*τ*_D _and *E *expected for the case of a single fixed distance (Eq. 6) and the broad distance distribution of a Gaussian chain [13], respectively. (e) Subpopulation-specific time-correlated single photon counting histograms for donor and acceptor photons from all bursts assigned to unfolded molecules, and subpopulation-specific donor intensity correlation function, in this case reporting on the nanosecond reconfiguration dynamics of the unfolded protein.

This requirement makes fluorescence a very attractive approach, where a dye molecule resonantly interacts with the excitation light [41-43]. Due to the Stokes shift, the emitted light can be selected spectrally from scattered light, e.g. with interference filters (Figure 2a), which can provide ratios of transmitted and reflected intensities at different wavelengths in excess of 10^6^. Since the background signal from solvent is proportional to the number of solvent molecules contained in the total observed volume, a common strategy for the detection of signal from individual molecules is to reduce the size of the detection volume as much as possible. Such spatial selection can be achieved, for instance, by very tightly focusing a laser beam into the sample, combined with confocal detection [44], or by total internal reflection fluorescence (TIRF) [45], which allows excitation of a ~200 nm layer of the sample in the evanescent field of a reflected laser beam. A further reduction of the observation volume can be achieved with zero mode waveguides, which allow measurements at higher concentrations of fluorescent sample [46]. Here, I will focus on confocal fluorescence detection because it is a commonly used method; currently allows the broadest range of accessible timescales; and enables the most complete access to the photon statistics of the fluorescence process.

### Fluorescence labelling

To be able to observe individual biomolecules, we first need to furnish them with suitable fluorophores. Since even tryptophan, the natural amino acid with the highest fluorescence quantum yield (~0.13), is not suitable for single molecule detection owing to its low photostability, and since fluorescent proteins [47] are still of limited use for the investigation of biomolecular structure and dynamics because of their relatively large size and their inferior photophysical properties, labelling with extrinsic fluorophores absorbing and emitting in the visible range of the spectrum is usually unavoidable for single-molecule spectroscopy. For FRET, two (or more) chromophores are needed, and their specific placement in the protein ideally requires groups with orthogonal chemistry. A wide range of labelling strategies exist, ranging from nonspecific labelling of amino or thiol groups present in the natural polypeptide [48] to the incorporation of non-natural amino acids [49], advanced chemical ligation [50], and intein-based approaches [51]. I refer the reader to reviews of the topic for a more detailed treatment [48,52,53].

Currently, the simplest and most common approach for labelling proteins is still to rely on cysteine derivatisation using maleimide chemistry. Increased specificity can be achieved by removing unwanted natural cysteines by site-directed mutagenesis or by introducing cysteines with different reactivity owing to different molecular environments within the protein [54]. Labelling is usually combined with multiple chromatography steps to purify the desired adducts. If higher specificity is required, e.g. for FRET with more than two fluorophores [55], cysteine labelling can be combined with orthogonal chemistries, such as non-natural amino acid incorporation [56]. A wide variety of suitable organic dyes with various functional groups for protein labelling have become commercially available. Examples of particularly popular chromophores for single molecule FRET are the cyanine dyes [57] or the Alexa Fluor series [58], which meet the key requirements for single-molecule fluorescence of biomolecules: high extinction coefficients and fluorescence quantum yields, high photostability, and sufficient solubility in aqueous solutions.

### Confocal single-molecule spectroscopy

Let us now assume that we have a suitably labelled molecule available, which carries at specific positions of our protein a donor and an acceptor fluorophore suitable for FRET (Figure 2b). In the simplest type of experiment, these molecules are diffusing freely in solution. In a confocal instrument [59], we achieve single-molecule detection in the following way (Figure 2a): a laser beam is focused into the sample solution to a diffraction-limited spot with a high numerical aperture objective. The small size of the resulting excitation volume (together with the confocal pinhole, see below) provides the requisite spatial selection, with an observation volume of ~1fl. Given the low concentration of fluorescently labelled molecules in solution (typically in the 10 to 100 pM range), the probability of two molecules residing in the confocal volume at the same time becomes negligible.

We use a laser wavelength for excitation that is resonant with the donor fluorophore of our FRET-labelled protein. If now a molecule happens to diffuse into the excitation volume, the donor dye will get electronically excited and can relax back to the ground state either by emitting a photon itself or by transferring its energy to the second fluorophore attached to our protein, the acceptor dye (Figure 2b). Owing to the pronounced distance dependence of coupling between donor and acceptor (Figure 1), the probability of energy transfer and thus the numbers of donor and acceptor photons emitted are highly sensitive to the interdye distance. In other words, we can obtain information about intramolecular distances by counting the numbers of photons emitted by donor and acceptor.

Emitted photons are collected by the same objective used for focusing the laser (epifluorescence). A dichroic mirror that reflects the laser light but transmits the emitted fluorescence, in combination with suitably chosen filters, provides the necessary *spectral separation *between fluorescence emitted by our labelled molecule and light scattered by the solvent (Figure 2a). A confocal pinhole in the image plane of the objective serves as a spatial filter to remove out-of-focus light and thus completes the *spatial selection *of the confocal detection scheme; except for a few background photons, we are now left only with fluorescence photons resulting from the FRET process. In the next steps, these photons are first sorted by their polarization using a polarizing beam splitter, and then by their wavelength, to distinguish donor and acceptor emission. The light is focused on highly sensitive detectors, typically avalanche photodiodes (APDs), where the individual photons trigger electronic pulses that are recorded by suitable counting electronics, in modern instruments with a time resolution limited by the jitter of the detectors, which is typically in the 50 ps range. In such an instrument, the time stamps of all detected photons, together with the corresponding detection channels, are stored for subsequent processing. If pulsed laser excitation is used, the time of the exciting laser pulse is also recorded.

### Basics of data analysis

From these records we thus have information on the colour, polarization, and arrival time of every individual photon, both on an absolute time scale and relative to the exciting laser pulse. The number of vertically and horizontally polarized photons can be used to calculate fluorescence polarization or anisotropy and thus reports on the rotational mobility of the fluorophores, a key aspect for quantitatively relating transfer efficiencies to distances [34]; the arrival time of a photon relative to the exciting laser pulse reports on the fluorescence lifetime of the fluorophore, a quantity that is complementary to ratiometric transfer efficiencies [60]; and the absolute arrival times can be employed to calculate correlation functions from picoseconds to seconds [61,62]. This type of data acquisition, which allows a wide range of derived quantities to be obtained from the data, is also termed multiparameter fluorescence detection [63].

A simple and common first step in analysing the data is to plot the number of donor and acceptor photons detected in a suitably chosen time bin, e.g. 1 ms. A typical result is shown in Figure 2c, where bins with very low signal (corresponding to the background) are interrupted by bursts of photons originating from individual molecules diffusing through the confocal observation volume. The duration of these fluorescence bursts is determined by the time it takes a molecule to diffuse through the confocal volume, which in many typical applications for biomolecules is on the order of 1 ms; during each burst, typically up to several hundred photons can be detected. The bursts can be identified by some threshold criterion (e.g. that more than 100 photons are detected in a millisecond bin, but more sophisticated algorithms are frequently employed [64]), and each of them can be analysed individually, e.g. in terms of the transfer efficiency of the respective molecule. The most widely used procedure is to calculate the transfer efficiencies ratiometrically [65] based on the number of detected photons from donor and acceptor, *n*_D _and *n*_A_, respectively:

(5) $$E=\frac{n_{A}}{n_{A}+n_{D}}.$$

(*n*_D _and *n*_A _need to be corrected for the differences in quantum yields of the dyes, the efficiencies of the detection system in the corresponding wavelength ranges, and related effects [48,65]. Note that *E *defined in this way is a randomly distributed quantity, see below. For the true transfer efficiency, the average photon *count rates *from donor and acceptor have to be used instead of *n_A _*and *n_D_*.) From a typical measurement (of minutes to hours, depending on the statistics required), thousands of such events are acquired and used to construct histograms (Figure 2d) that represent the distribution of transfer efficiencies present in the sample.

Figure 2d illustrates such a histogram in terms of a simple example. A small FRET-labelled protein is investigated under conditions where both the folded and the unfolded state are populated at equilibrium (in this example at 1.5 M of the denaturant guanidinium chloride, GdmCl). In the folded structure, donor and acceptor are in close proximity, resulting in high transfer efficiency values; in the unfolded state, the average distance between the fluorophores is much greater, and the transfer efficiency is thus expected to be lower. Two corresponding peaks are observed in the transfer efficiency histogram. This aspect already illustrates one of the key strengths of single molecule spectroscopy: the two subpopulations present in the sample can be separated and thus investigated individually even if they coexist. The corresponding ensemble experiment would only provide the average over the entire transfer efficiency distribution, and extracting information about the subpopulations would be much more difficult or even impossible. An additional peak is usually observed at a transfer efficiency of zero. This "donor-only" peak results from molecules that lack an active acceptor, either owing to imperfections of the labelling procedure or because the acceptor dye was photobleached in a previous passage through the confocal volume. This simple analysis provides a first impression of the heterogeneity of the sample and the number of subpopulations present.

However, with the availability of the other parameters mentioned above, we can also proceed from one-dimensional to higher-dimensional data analysis. Figure 2d shows an example where the relative fluorescence lifetime of the donor is plotted versus the transfer efficiency in a 2D histogram. The donor fluorescence lifetime provides an independent way of determining the transfer efficiency that is less susceptible to artefacts (caused e.g. by fluorescence quenching) than the ratiometric measurement [63]. In the simplest case of a single fixed distance, the fluorescence lifetime of the donor in the presence (*τ*_DA_) and absence (*τ*_D_) of the acceptor can be related to the transfer efficiency by [34]

(6) $$E=1-\frac{\tau_{DA}}{\tau_{D}}.$$

(If distance distributions are present in the molecules, this simple relationship no longer holds, and the experimentally observed deviations can be used to infer information about the distance distribution and the underlying dynamics [63,66], see Figure 2d.) Similarly, plots involving fluorescence anisotropies can be used to assess the rotational mobility of the dyes and judge whether orientational averaging of the chromophores can be assumed, which greatly simplifies the FRET analysis, or whether their relative orientational distribution has to be taken into account explicitly [34,67]. All of these parameters can be calculated from the signal of each individual molecule [64], be it in free diffusion experiments or in measurements on immobilized samples. Note, however, that determining quantities such as the transfer efficiency or the fluorescence lifetime from ~100 photons is intrinsically uncertain, as will be discussed below. This uncertainty can be reduced by subpopulation-specific analysis. If, e.g., all photons from bursts originating from unfolded molecules are combined, fluorescence intensity decays for the unfolded state can be extracted with much better signal-to-noise ratio (Figure 2e). Similarly, subpopulation-specific correlation functions can be extracted to investigate the dynamics of the molecules in a specific state or conformation (Figure 2e).

### Variations on the theme

The procedures outlined so far represent a common approach in advanced single-molecule fluorescence spectroscopy, and suitable four-channel instruments are commercially available [68]. However, there are many variants of the type of instrumentation and analysis methods, which cannot be discussed here in detail. Instead I will mention some of them briefly and refer the reader to examples from the relevant literature.

For basic applications, simpler instruments with only two detection channels and continuous-wave laser excitation are very commonly employed. Such instruments are sufficient to measure ratiometric transfer efficiencies, but they lack the possibility of extending the analysis to fluorescence lifetimes or anisotropies at the same time. Another possibility available with more than two detections channels is to sort the photons by more than two wavelength bands [63]. A powerful application is the use of multicolour-FRET, where more than two fluorophores are used, which in principle allows three and more distances to be determined simultaneously [69-71]. A common technique is the use of multiple lasers for probing both donor and acceptor dye [72,73]. By rapidly alternating donor and acceptor excitation on the nanosecond to microsecond timescale, donor and acceptor dye can be probed independently, and molecules with low transfer efficiency can be distinguished from "donor-only" events. Additionally, the stoichiometries of donor to acceptor dyes present in the molecules can be quantified, which is often of interest for intermolecular interactions [56,57]. The types of lasers employed also vary. For some experiments, the use of pulsed lasers is advantageous because fluorescence lifetimes can be determined. For the measurement of sub-microsecond dynamics with correlation spectroscopy (see below), however, continuous wave lasers are employed. The choice of fluorophores will define the wavelengths required for excitation.

A popular setup complementary to confocal detection is wide-field microscopy with two-dimensional detectors such as sensitive CCD cameras, often in combination with total internal reflection fluorescence (TIRF) [1,45]. Wide-field imaging has the big advantage that it allows the collection of data from many single molecules in parallel. However, it requires their immobilization at the surface for excitation by the shallow evanescent field that is generated by the reflected laser beam. The time resolution of wide-field methods is currently limited to the millisecond range by the frame-transfer rates of cameras with sufficiently high detection efficiency. This approach is thus most commonly applied to the investigation of processes on timescales of seconds to minutes. Note that confocal detection also allows the interrogation of surface-immobilized molecules, but the molecules have to be probed serially by sample or laser scanning, which often complicates experiments under non-equilibrium conditions.

Finally, a wide variety of data analysis methods beyond the basic set of commonly used procedures are employed. These range from different strategies for burst identification (e.g. binning with fixed time windows or based on inter-photon times [60]), correction for background, direct excitation, and crosstalk between the detection channels [48,63], to a wide range of more sophisticated methods that take into account the relationship between all recorded parameters [63] and the details of photon statistics [18,74-78].

### Photon statistics and FRET efficiency distributions

A detailed treatment of the role of photon statistics for the analysis of single-molecule data is beyond the scope of this basic tutorial, but a few aspects are essential to keep in mind. First, it is important to recognize that even for a molecule with a single fixed distance, the resulting FRET efficiency histogram is broadened. A fundamental source of broadening is shot noise, the variation in count rates about their mean values due to the discrete nature of the signal. In other words, we observe a statistical distribution of FRET efficiencies since only small numbers of photons are detected from an individual molecule. The variance of the corresponding transfer efficiency distribution due to shot noise can be calculated as [79]

(7) $$\sigma_{sn}^{2}=\left\langle E^{2}\right\rangle -{\left\langle E\right\rangle }^{2}=\left\langle E\right\rangle \left(1-\left\langle E\right\rangle \right)\left\langle 1/N\right\rangle \;\leq \left\langle E\right\rangle \left(1-\left\langle E\right\rangle \right)/N_{T},$$

where 〈*E*〉 is the true underlying mean transfer efficiency, 〈1/*N*〉 is the average of the inverse numbers of photons per burst, and *N_T _*is the minimum burst size, i.e. the threshold used for data analysis. In other words, the observation of a broadened transfer efficiency histogram does not necessarily imply the existence of a broad underlying distance distribution. Similar considerations lead to broad distributions of fluorescence lifetimes or anisotropies from observations involving a small number of photons.

Since the contribution of shot noise to the measured transfer efficiency distributions is well understood, rigorous methods are available to fit the measured transfer efficiency histograms directly assuming an underlying, shot noise-free, transfer efficiency distribution [74,79-82]. In the case of a single fixed distance (or dynamics much faster than the interphoton time, see below), this resulting distribution should be close to a delta function. In practice, histograms broader than expected from shot noise alone are commonly observed. The origin of this excess width is often difficult to establish unequivocally [17,83], but there are factors besides conformational heterogeneity that can contribute, such as variations in fluorescence quantum efficiencies, labelling permutations, or an imperfect alignment of the confocal volumes for donor and acceptor channels. Consequently, without a suitable reference, it is often difficult to interpret a width in excess of shot noise in terms of slow conformational dynamics. Multiparameter fluorescence detection is very helpful for identifying such complications and taking them into account for the analysis [63].

The most interesting cause of broadening of the transfer efficiency distribution is of course the presence of a distance distribution in the sample. In some cases, this heterogeneity is quite obvious, as in the case of folded and unfolded molecules shown in Figure 2d. However, take for example the subpopulation of unfolded molecules itself: clearly, a broad distribution of intramolecular distances will be present in the unfolded state, and yet, the peak corresponding to unfolded molecules is not much broader than that of the folded state. As in many other spectroscopic techniques, a crucial aspect that needs to be taken into account are the timescales involved in the measurement and the dynamics of the molecular system.

### Distance distributions and relevant timescales

The relative magnitudes of the timescales of at least four different processes have an influence on the position and the width of the FRET efficiency histogram: (a) the rotational correlation time of the chromophores, (b) the fluorescence lifetime of the donor, (c) the intramolecular dynamics of the molecule probed by the fluorophores, and (d) the observation timescale.

The rotational correlation time of the chromophores influences the value of the orientation factor *κ*^2 ^(Eq. 3): if dye reorientation is sufficiently fast for the relative orientation of the donor and acceptor dipoles to average out while the donor is in the excited state, *κ*^2 ^can be assumed to equal 2/3. If, in the other extreme, the donor fluorescence decay is much faster than dye reorientation, a static distribution of relative dye orientations can be assumed. Intermediate cases are difficult to treat analytically [34], and simulations become the method of choice. *κ*^2 ^= 2/3 is often a good approximation because the rotational correlation times of typical dyes are in the range of a few hundred picoseconds (if there is no persistent interaction with the protein), while their fluorescence lifetimes are in the nanosecond range (although they may be reduced significantly by the transfer process), but this aspect must be verified for every molecular system under investigation.

**Figure 3:**
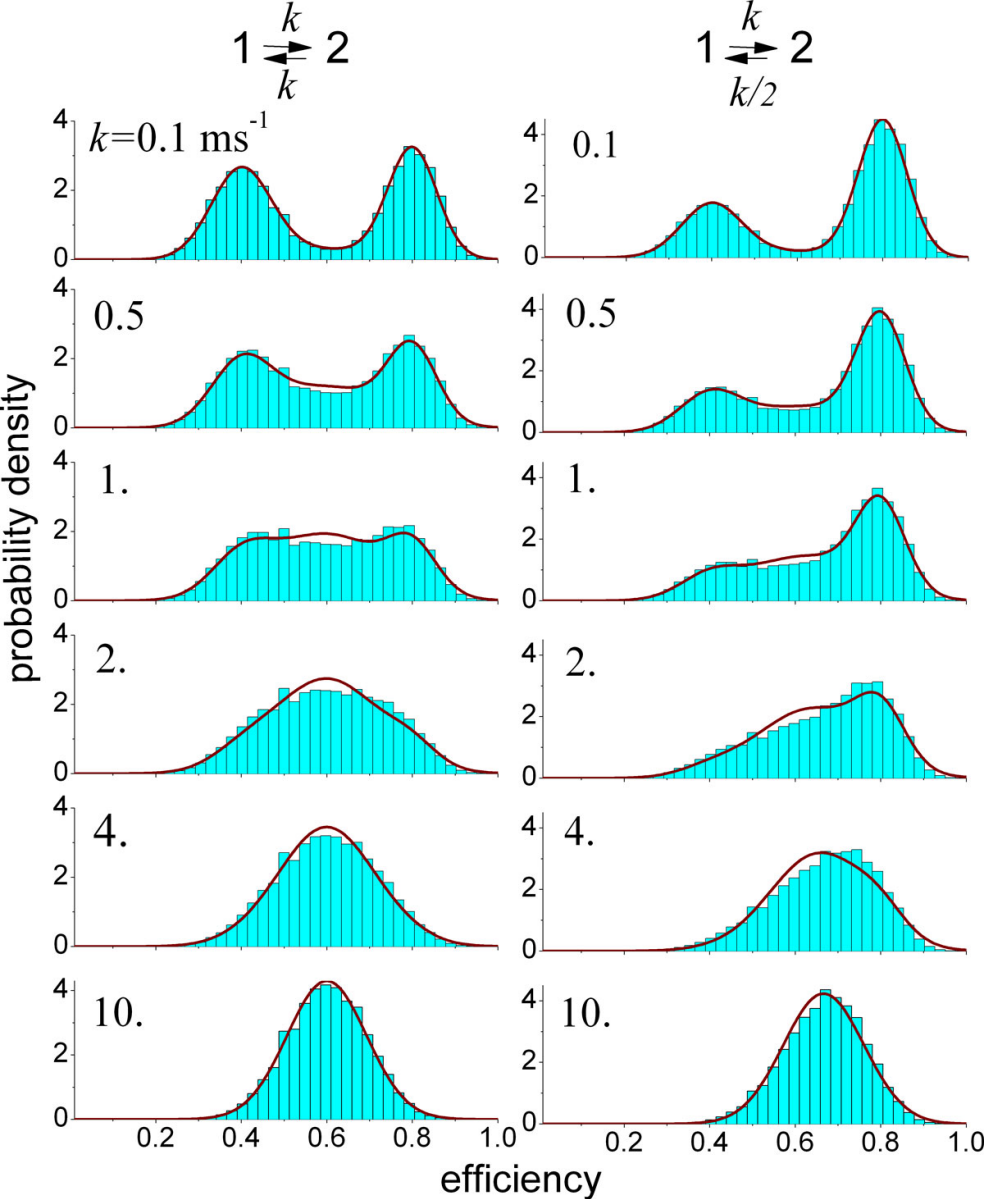
**Effects of conformational dynamics on transfer efficiency histograms in single-molecule FRET experiments on freely diffusing molecules**. The histograms (blue bars) were calculated assuming that the molecules in the sample undergo transitions between two conformational states with low (*E*_1 _= 0.4) and high (*E*_2 _= 0.8) transfer efficiency. The histograms are shown for different values of the transition rate, given in the upper left corner of the histogram. In the first column, the equilibrium populations of two the states are equal; in the second column, the populations differ by a factor of 2. For low values of *k*, two well-separated peaks are observed, corresponding to the two subpopulations. With increasing relaxation rate, it becomes more likely that a conformational transition occurs during a transit of the molecule through the confocal volume (and thus a fluorescence burst), resulting in an increasing number of events with apparent transfer efficiencies in the range between the true transfer efficiencies *E*_1 _and *E*_2_. At high relaxation rates, transitions occur so frequently during a fluorescence burst that the two subpopulations become indistinguishable; only one peak remains, whose relative position between *E*_1 _and *E*_2 _is determined by the equilibrium constant. The histograms are compared to the Gaussian approximation [20] (red lines), which can also be used to determine rate constants from measured histograms[19]. Figure provided by Irina Gopich [79].

Most importantly, the timescale of interdye distance dynamics relative to the observation time (more accurately, the inter-photon times [84]) will affect the width of the measured transfer efficiency distributions. As shown by Gopich and Szabo [80,84], the observation time must be approximately an order of magnitude smaller than the relaxation time of the donor-acceptor distance to obtain physically meaningful distance distributions or corresponding potentials of mean force from transfer efficiency histograms. Otherwise, only the mean value of the transfer efficiency of the respective subpopulation can be used to extract information about the distance distribution, and an independent model for the shape of the distance distribution is needed. In practice, this means that distance distributions can be determined from FRET efficiency histograms from free diffusion experiments if the underlying dynamics are on a timescale greater than about 1 ms, assuming photon count rates of ~10^5 ^s^-1 ^typically achieved during fluorescence bursts [84]. A noticeable influence of dynamics on the width of the transfer efficiency histograms, however, is already expected for fluctuations in the 10 to 100 *μ*s timescale [17,84]. As a result, reaction dynamics can be obtained from the shape of transfer efficiency histograms [19,85]. Figure 3 illustrates how changes in the interconversion kinetics between two populations can influence the shape of FRET efficiency histograms and how their analysis can be used to determine the underlying exchange kinetics [20,76].

Given a distance distribution, *P*(*r*), three physically plausible limits for the possible averaging regimes and the resulting mean transfer efficiencies, 〈*E*〉, are [86,87]:

1. If the rotational correlation time, *τ*_c_, of the chromophores is small relative to the fluorescence lifetime, *τ*_D_, of the donor (i.e. *κ*^2 ^= 2/3), and the dynamics of the polypeptide chain (with relaxation time *τ*_*r*_) are slow relative to *τ*_D_,

(8) $$\left\langle E\right\rangle =\int_{a}^{l_{c}}E\left(r\right)P\left(r\right)\;dr\;\text{with}\;E\left(r\right)\;\;\;=\;\;\;{\left(1\;\;+\;\;{\left(r/R_{0}\right)}^{6}\right)}^{-1},$$

where *P*(*r*) is the normalized inter-dye distance distribution; *a *is the distance of closest approach of the dyes; and *l*_c _is the contour length of the peptide. This scenario typically applies to cases such as unfolded or intrinsically disordered proteins [12,17,88].

2. If *τ*_c _≪ *τ*_D _and *τ*_*r *_≪ *τ*_D_,

(9) $$\left\langle E\right\rangle =\frac{\int_{a}^{l_{c}}{\left(R_{0}/r\right)}^{6}P\left(r\right)\;dr}{1+\int_{a}^{l_{c}}{\left(R_{0}/r\right)}^{6}P\left(r\right)\;dr}.$$

This dynamic averaging regime often applies to the local motion of the dyes on their linkers or in short peptides [87].

3. If *τ*_c _≫ *τ*_D _and *τ*_*r *_≫ *τ*_D_,

(10) $$\left\langle E\right\rangle =\int_{0}^{4}\int_{a}^{l_{c}}E\left(r,\kappa^{2}\right)\;P\left(r\right)\;p\left(\kappa^{2}\right)\;dr\;d\kappa^{2}\;\text{with}\;E\left(r,\kappa^{2}\right)=\;\;\;{\left(1\;\;\;+\;\;\;\frac{2}{3\kappa^{2}}{\left(r/R_{0}\right)}^{6}\right)}^{-1}.$$

This limit would correspond to a situation where both the relative orientations of the fluorophores and the distance distribution are essentially static on the observation timescale. In such a scenario, extreme broadening of the transfer efficiency distributions can result, with contributions from both orientational and distance effects on the FRET efficiency [34,67]. The theoretical isotropic probability density of *κ*^2 ^for the case in which all orientations of the donor and acceptor transition dipoles are equally probable is [34,89]

(11) $$p\left(\kappa^{2}\right)\;\;\;=\{\begin{array}{c} \frac{1}{2\sqrt{3\kappa^{2}}}\ln\left(2\;+\;\sqrt{3}\right)\;\;\;\;\;\;\;\;\;\;\;\;\;\;\;\;\;\;\;\;\;\;\;\;\;\;\;\;\;\;\;0\;\;\leq \;\;\kappa^{2}\;\;\leq \;\;1\\ \frac{1}{2\sqrt{3\kappa^{2}}}\;\ln\left(\frac{2\;+\;\sqrt{3}}{\sqrt{\kappa^{2}}\;\;+\;\;\sqrt{\kappa^{2}\;-1}}\right)\;\;\;\;\;\;\;\;\;\;\;1\;\;\leq \;\;\kappa^{2}\;\;\leq \;\;4\;\; \end{array}.$$

Another interesting consequence of the limiting cases above can be illustrated with the example of an unfolded protein (Figure 2). As will be shown in detail below, the inter-dye distance dynamics in this case are much faster (~100 ns) than the millisecond observation time and the average interphoton time relevant for the transfer efficiency histogram. As a result, the width of the histogram does not report on the distance distribution, but only on its mean value - the information on the shape of *P*(*r*) is lost. The fluorescence lifetime (~1 ns), however, is much shorter than the timescale of chain dynamics. In other words, the chain is essentially static during an excitation/emission cycle of the fluorophores, and the distribution of transfer rates resulting from the broad inter-dye distance distribution will give rise to highly non-exponential fluorescence decays, which contain information on the shape of *P*(*r*) [37]. From single-molecule FRET measurements, these decays can be determined for individual subpopulations to remove contributions from other species (Figure 2d, e) and can be analysed in detail in terms of distance distributions [88,90]. As a result, the presence or absence of a distance distribution will also affect the position and shape of the subpopulation peaks in lifetime vs. transfer efficiency 2D-histograms (Figure 2d) [13,66,78].

These examples illustrate how the timescales of the FRET process and the molecular dynamics influence the transfer efficiencies and their distributions observed in single-molecule experiments. For a quantitative analysis of single-molecule FRET experiments, it is thus essential to know the relevant timescales involved. How fluorescence lifetimes and anisotropies can be obtained from the measurements was already discussed, and these are essential for quantifying *τ*_D _and *τ*_c_, and thus for assigning the experimental situation in question to the limiting cases given above. Another very generally applicable method for quantifying the dynamics of the system is correlation spectroscopy.

### Correlation analysis

In ensemble experiments, the most common approach for investigating the relaxation kinetics of a reaction is to perturb the entire sample, e.g. by a rapid change in denaturant concentration in a stopped-flow instrument or by a laser-induced temperature jump, and then to observe the system return to equilibrium under the new set of conditions. The resulting kinetics can be analysed in terms of kinetic models to identify the underlying molecular mechanisms. According to the fluctuation-dissipation theorem [91], the rate of relaxation of a system to equilibrium after a small macroscopic perturbation and the time correlation of spontaneous fluctuations of the undisturbed system at equilibrium are described by the same rate coefficients. Single-molecule spectroscopy allows us to detect such spontaneous fluctuations at equilibrium, and correlation analysis is a versatile approach for quantifying the dynamics of chemical reactions or conformational changes in the absence of perturbations.

For instance, the number of molecules present in a confocal volume will fluctuate since molecules continuously enter and leave the observation region by diffusion. Similarly, if we consider the example of a folding protein, each molecule will stochastically jump between folded and unfolded states. Both processes will lead to fluctuations in the fluorescence count rates. Powerful tools for analysing such fluctuations are correlation functions [92]. The autocorrelation function of the property *A*, for instance, is defined as

(12) $$\left\langle A\left(t\right)A\left(t+\tau \right)\right\rangle =\underset{T\to \infty }{\lim}\frac{1}{T}\overset{T}{\underset{0}{\int }}A\left(t\right)A\left(t+\tau \right)\;dt.$$

(Strictly speaking, this definition is only true for an ergodic system, where the averaging is independent of the starting time *t*, and in any experimental measurement the averaging is of course done over finite time.) Crosscorrelation functions between different properties or signals, e.g. fluorescence emission from a donor and an acceptor chromophores undergoing FRET, can be defined analogously. The autocorrelation function of a non-conserved, non-periodic property decays from its initial value 〈*A*^2^〉 to the final value 〈*A*〉^2 ^with a time constant characteristic of the fluctuation of *A*, where *A*(*t*) and *A*(*t*+*τ*) are expected to become uncorrelated at long times. In many cases, the autocorrelation function decays like a single exponential with a characteristic *relaxation time *or *correlation time *of the property, but often it takes a more complicated functional form.

### Fluorescence correlation spectroscopy (FCS)

**Figure 4:**
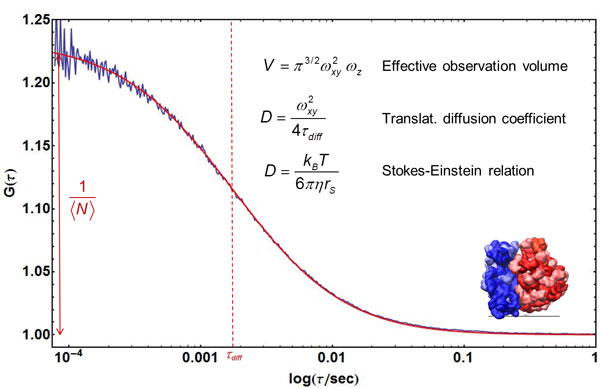
**Quantifying translational diffusion with FCS**. An FCS curve of fluorescently labelled ribosomes freely diffusing in solution (blue) is shown with a fit using Eq. 13 (omitting the exponential term; data by J. Clark & B. Schuler, unpublished). From the amplitude of the correlation function, the average number of fluorescent particles in the confocal volume, 〈*N*〉, can be determined. With knowledge of the size of the confocal volume, *V*, based on its half-width, *ω_xy_*, and height, *ω_z_*, the particle concentration can be calculated. From the diffusion time, *τ_D_*, the translational diffusion coefficient, *D*, of the particles is obtained, which can be related to the Stokes radius, *r_s_*, via the Stokes-Einstein relation (where *k_B _*is Boltzmann's constant, *T *is temperature, and *η *is the solution viscosity). The inset shows a structural representation of the ribosome (*r_s _*≈ 12 nm).

A common example for a fluorescence correlation experiment [93] is the measurement of translational diffusion, where fluorescently labelled molecules diffuse through a confocal volume approximated by a three-dimensional Gaussian shape. The resulting intensity autocorrelation function normalised by the mean intensity squared is [27]

(13) $$G\left(\tau \right)=1+\frac{1}{\left\langle N\right\rangle }\;\;{\left(1+\frac{\tau }{\tau_{diff}}\right)}^{-1}{\left(1+\frac{\tau }{\omega^{2}\;\tau_{diff}}\right)}^{-1/2}\left(1+K\;e^{-\tau /\tau_{r}}\right),$$

where 〈*N*〉 is the average number of molecules in the observed volume; *τ*_*diff *_is the characteristic time it takes a molecule to diffuse through the observation volume; *ω *is the aspect ratio of the volume; and *K *is the equilibrium constant of a reaction with a relaxation time *τ*_r _resulting in fluctuations of the emission intensity. In this case, there are thus two mechanisms contributing to the observed intensity fluctuations: diffusion of molecules in and out of the confocal volume, and fluctuations in the fluorescence rates caused by the reaction, which could, for example, be a protein folding reaction. (From Eq. 13, it is obvious that the observation of reaction dynamics is limited to timescales not much greater than the diffusion timescale, because the diffusive terms will decay to zero for *τ *≫ *τ*_*diff*_.) Fits of corresponding experiments with Eq. 13 can thus be used to determine several parameters: translational diffusion coefficients from *τ*_*diff *_and the radius of the confocal volume (Figure 4); the concentration of fluorescent molecules from 〈*N*〉 and the size of confocal volume (Figure 4); and the equilibrium constant and relaxation dynamics of the reaction. Note that for FCS experiments, the sample concentration is typically chosen in the nanomolar range to optimize the signal-to-noise ratio [27]. In this concentration range, a separation of fluorescence bursts from background signal is no longer possible with usual confocal instruments; however, the same analysis can of course be applied to picomolar solutions (Figure 2c, e) and thus be combined with the single-molecule methods described above.

**Figure 5:**
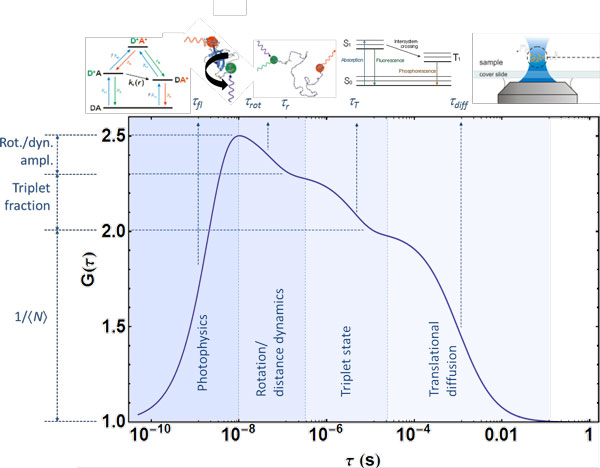
**Correlation spectroscopy can be used to probe a wide range of timescales and processes**. Illustration of processes that can contribute to the fluorescence correlation functions of freely diffusing molecules, with their characteristic timescales and corresponding amplitudes. Illustrated are: photon antibunching [112] in the range of the fluorescence lifetimes of the fluorophores, *τ_fl_*; rotational diffusion with a correlation time *τ_rot_*, and interdye distance dynamics due to FRET with a reconfiguration time *τ_r _*(note that *τ_rot _*and *τ_r _*can be in a similar range, but can be distinguished by the donor-acceptor crosscorrelation (Figure 6), which shows anticorrelated behaviour in the case of distance dynamics); triplet state blinking on a timescale *τ_T_*; and translational diffusion (Figure 4) with a diffusion time *τ_diff_*. Timescales much greater than *τ_diff _*are not accessible with freely diffusing molecules, but can be extended by taking advantage of recurrence effects [18] (i.e. molecules returning to the confocal volume) or with experiments on immobilized molecules (Figure 7).

More generally speaking, any process that leads to fluctuations in fluorescence count rates on an accessible timescale will contribute to the shape of the correlation function (Figure 5). Besides translational diffusion and reaction dynamics, such processes can be of photophysical origin (e.g. triplet state blinking in the microsecond range or photon antibunching [94] resulting from the property of a single quantum system that it cannot emit two photons at the same time, and which thus occurs in the range of the fluorescence lifetime); they can result from rotational diffusion (typically on the 1 to 100 ns timescale); or from molecular interactions between different fluorescently labelled species, to name a few examples.

**Figure 6:**
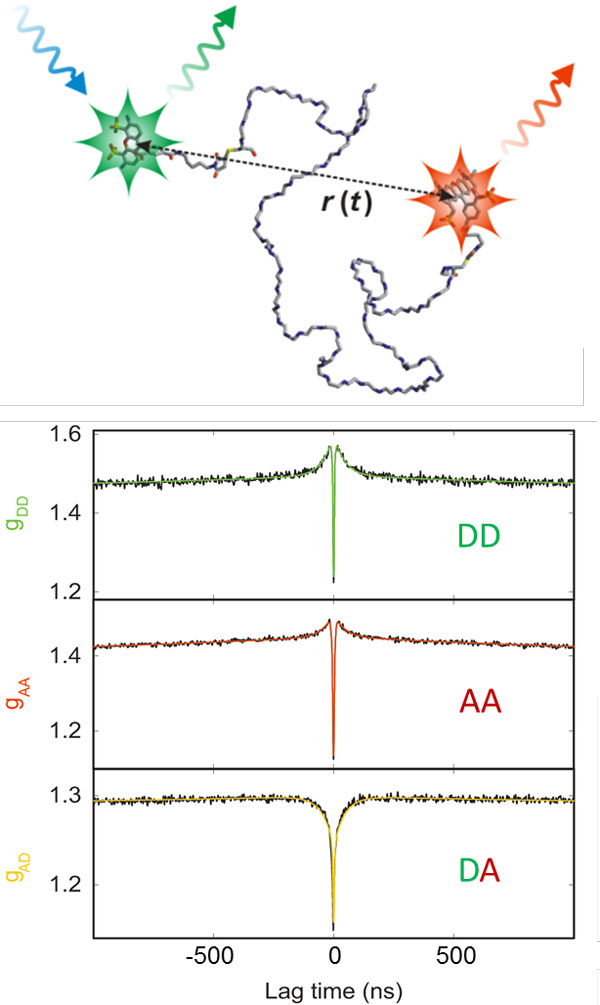
**Nanosecond correlation spectroscopy used for the determination of unfolded state dynamics on the sub-microsecond timescale**. (top) Cartoon of a FRET-labelled unfolded protein undergoing diffusive chain dynamics, resulting in fluctuations of the inter-dye distance, *r*(*t*) [12]. (bottom) Donor-donor (DD), acceptor-acceptor (AA), and donor-acceptor (DA) intensity correlation functions for an unfolded protein are shown (Csp*Tm *in 4 M GdmCl [96]) in the nanosecond range, where the decay at about 50 ns reports on the reconfiguration time of the polypeptide chain. Note that the donor-acceptor crosscorrelation shows anticorrelated behaviour, a signature of distance dynamics. Antibunching [112] occurs on the timescale of a few nanoseconds. Fits are shown as solid lines [96].

Of particular interest for the investigation of biomolecules are fluorescence fluctuations caused by conformational dynamics. An example that illustrates the possibility to extract very rapid dynamics from correlation spectroscopy is given in Figure 6. Consider an unfolded polypeptide chain labelled with a FRET pair. In solution, the chain will show rapid diffusive intramolecular dynamics, and as a result, the distance between donor and acceptor will fluctuate. Owing to the FRET coupling between them, donor and acceptor emission rates will fluctuate on the same timescale. This timescale characterises the intramolecular dynamics of the chain and closely corresponds to its reconfiguration time [95]. By analysing the donor and acceptor intensity correlation functions, the reconfiguration time can be quantified; for unfolded and intrinsically disordered proteins it turns out to be in the range of ~100 ns [12,13,96-98], close to the reconfiguration times expected from simple polymer dynamics [99], but with clear indications for the presence of "internal friction", i.e. nonspecific interactions within the chain that slow down the dynamics and depend on the compactness of the polypeptide chain [13,97].

Correlation analysis of fluorescent molecules freely diffusing in solution, as in typical FCS experiments, can provide information on dynamics from nanoseconds to milliseconds. However, correlation analysis can equally be applied to the signal recorded from single immobilised molecules to extend the range of accessible times. For immobilized molecules, the observation time is limited only by photobleaching, and with suitable photoprotective solution additives [100], observation times of minutes can be achieved [1]. The bleaching time will critically depend on the laser power and thus the excitation rates employed. Every experiment will thus have to be optimized with respect to the compromise between time resolution - determined by the number of photons detected per time - and the total observation time required to probe the dynamic processes of interest.

### Kinetics and thermodynamics from single-molecule fluorescence trajectories

**Figure 7:**
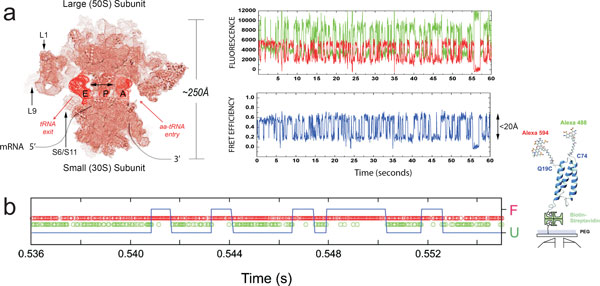
**Kinetics from single-molecule fluorescence trajectories and single-photon time series**. (a) A representation of a ribosome structure with different structural features and labelling sites is shown on the left. On the right, a single-molecule fluorescence trajectory (green, donor; red, acceptor) and resulting FRET trajectory (blue) obtained using a wide-field TIRF imaging system at 40 ms time resolution under equilibrium conditions from a surface-immobilized ribosome bearing site-specifically labelled A-site and P-site tRNA molecules [113]. The dynamic FRET data, reporting directly on thermally accessible, nanometre-scale changes in tRNA position within the ribosome, persist until donor and/or acceptor fluorophore photobleach. Figure from ref. [114]. (b) On the left, a photon time series (green, donor; red, acceptor) from a confocal measurement using single-photon counting is shown. A state trajectory obtained from the Viterbi algorithm is shown as a blue line, with segments corresponding to folded and unfolded protein, and transition points [19]. On the right, a cartoon of the structure of the small protein *α*_3_D immobilized on a surface is shown, whose folding was investigated here. Figure adapted from ref. [19].

In many cases, dynamics can be determined from single-molecule experiments without having to resort to correlation analysis. The most prominent examples involve dynamics on the timescale of seconds that are visible directly in fluorescence recordings of immobilized molecules, e.g. as jumps between two or more levels of transfer efficiencies, corresponding to different conformations of a molecule [1] (Figure 7a). With data of sufficient quality, the kinetics of the system can be analysed directly from the distributions of dwell times in the individual states, much alike the methods established in the field of single-channel recording [101]. In the simplest case of a two-state reaction, A ↔ B, the rate coefficients of interconversion can be obtained from the inverse values of the average dwell times in states A and B, respectively, and the equilibrium constant is given by the ratio of the average dell times [101]. In cases where the transitions are difficult to identify, hidden Markov models [25,102,103] or maximum likelihood methods [22,75,103] can be essential for a quantitative analysis in terms of kinetic models (Figure 7b).

### Nonequilibrium dynamics of single molecules

**Figure 8:**
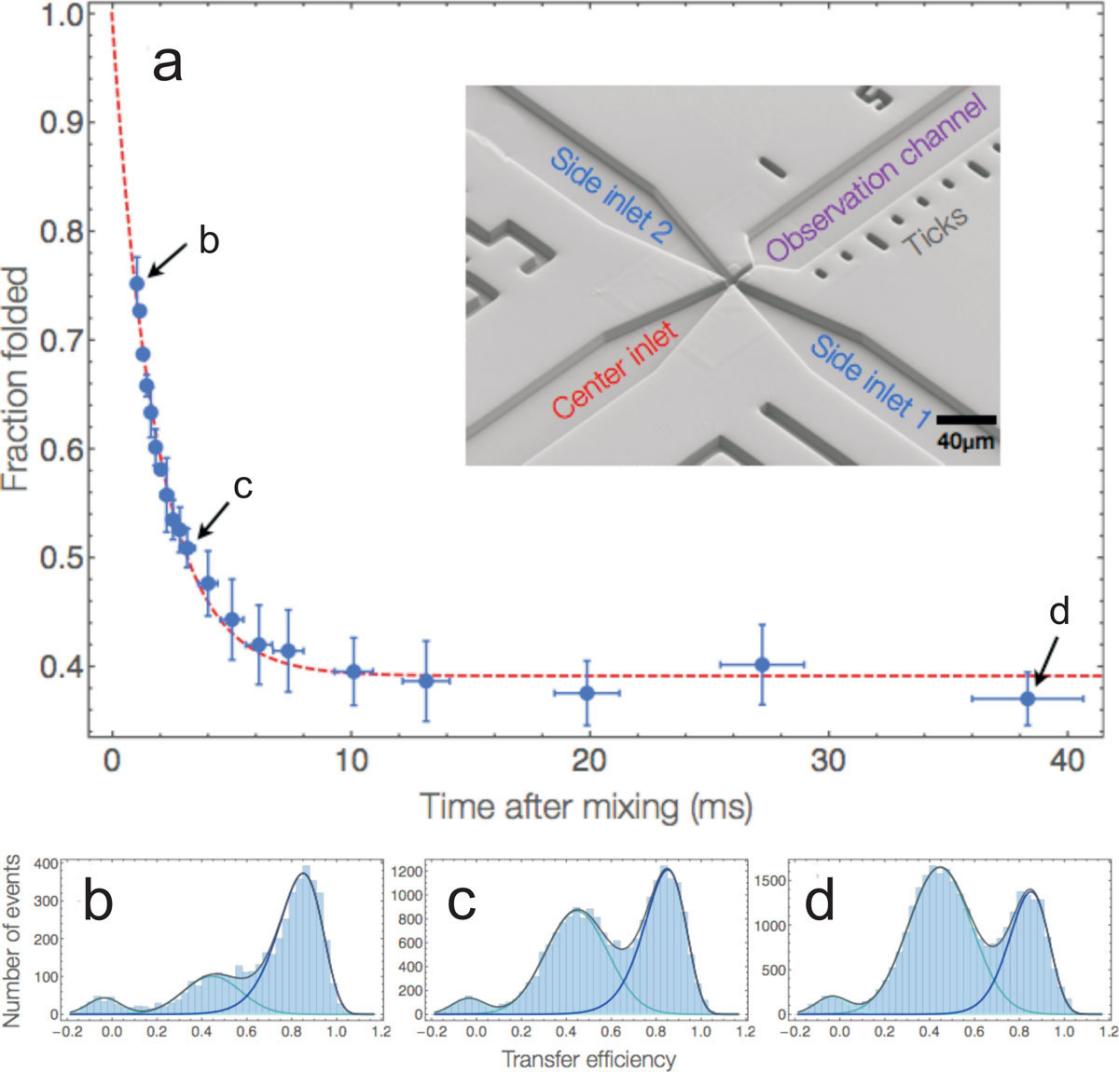
**Microfluidics for probing nonequilibrium dynamics with single-molecule spectroscopy**. The millisecond unfolding kinetics of a small protein (BdpA) upon mixing with denaturant are shown as an example for the application of microfluidics [115]. Folded BdpA in the centre inlet was mixed with 3.3 M of the denaturant GdmCl from the side inlet channels to trigger unfolding (see inset for an electron micrograph of the microfluidic structure in the mixing region of the device). (a) The fraction of folded population (blue data points) was determined from fits to the corresponding transfer efficiency histograms (solid lines in b-d) and plotted as a function of time after mixing. The data were fit with a single exponential decay (dashed red line). (b-d) Representative transfer efficiency histograms measured at the positions indicated in (a). Figure adapted from ref. [115].

Even though kinetic information can often be obtained from equilibrium single-molecule experiments, in many cases it is still essential to probe nonequilibrium dynamics, especially if the reaction of interest is essentially irreversible during the observation time accessible at equilibrium. A method that lends itself very well to the combination with single-molecule detection optics is microfluidic mixing, and several different implementations have been reported [104-109]. The basic idea of these devices is to mix solutions in stationary laminar flow by reducing the dimensions of the channels in order that the components of the solutions that are combined exchange very quickly, solely by diffusion [110]. After mixing, the confocal observation volume is placed at different points in the observation channel, corresponding to different times after the start of the reaction (Figure 8), with dead times in the millisecond range [109] and even below [107]. Microfluidic mixing can be used, e.g., to rapidly change solution conditions or to investigate the kinetics of protein-protein interactions [104-107,111] and has thus become a valuable extension of the growing single-molecule toolbox.

## Conclusions

I hope that this brief introductory tutorial will stimulate the reader's appetite for single-molecule spectroscopy and will facilitate the exploration of the more specialized literature. The rapidly growing number of applications of single-molecule spectroscopy to biomolecular systems now covers an impressive range of advanced techniques and topics, many of which could not even be mentioned here. Furthermore, even though single-molecule spectroscopy has reached a certain level of maturity, new techniques and analysis methods are still emerging on a regular basis, and the scope of single-molecule methods thus keeps expanding.

## List of abbreviations used

FRET: Förster resonance energy transfer; FCS: Fluorescence correlation spectroscopy; GdmCl: Guanidinium chloride.

## Competing interests

The author declares that he has no competing interests.
